# Statin Therapy and Symptom Burden in Patients With Fibromyalgia: A Prospective Questionnaire Study

**DOI:** 10.1016/j.mayocpiqo.2021.10.001

**Published:** 2021-11-01

**Authors:** Ryan S. D’Souza, Mary O. Whipple, Ann Vincent

**Affiliations:** a Department of Anesthesiology and Perioperative Medicine, Division of Pain Medicine; b Department of Medicine, Mayo Clinic, Rochester, MN; c Division of Geriatric Medicine, University of Colorado Anschutz Medical Campus, Aurora

**Keywords:** BMI, body mass index, FIQ-R, Revised Fibromyalgia Impact Questionnaire, FM, fibromyalgia, GAD-7, Generalized Anxiety Disorder 7-item, MASQ, Multiple Ability Self-Report Questionnaire, MFI-20, Multidimensional Fatigue Inventory 20-item, PHQ-9, Patient Health Questionnaire 9-item, SPI-II, Sleep Problems Index II

## Abstract

**Objective:**

To evaluate the association between statin use and symptom severity, tender point count, fatigue, cognition, mood, and sleep issues in patients with fibromyalgia (FM).

**Methods:**

Between May 2012 and November 2013, 668 patients with FM were surveyed. Patients were stratified into statin users and statin nonusers. Primary outcome was FM symptom severity (FIQ-R questionnaire) and tender point count. Secondary outcomes included fatigue (MFI-20), cognitive dysfunction (MASQ), anxiety (GAD-7), depression (PHQ-9), and sleep issues (SPI-II). Regression analysis assessed for differences in these clinical outcomes between statin users and statin nonusers and adjusted for age, sex, body mass index, ethnicity, tobacco use, opioid use, and neuropathic medication use.

**Results:**

Of the FM patients, 79 (11.8%) were statin users, whereas 589 (88.2%) reported no current statin use. Compared with the control cohort, statin users were older (55.0±11.3 years vs 46.2±12.9 years; *P*<.001) and had a higher body mass index (33.0±7.0 kg/m^2^ vs 29.8±7.7 kg/m^2^; *P*=.001). Adjusted linear regression revealed no association between statin use and symptom severity (total FIQ-R scores, 57.7±18.3 vs 59.0±18.1; adjusted β coefficient, −0.4; 95% CI, −4.8 to 4.1; *P*=.871). There was also no association between statin use and tender point count (14.8±4.1 vs 14.5±4.2; adjusted β coefficient, 0.2; 95% CI, −0.8 to 1.2; *P*=.732). Secondary outcome analysis revealed no difference between statin users and statin nonusers in metrics measuring fatigue, cognition, anxiety, depression, and sleep problems.

**Conclusion:**

Administration of statin therapy for at least 1 month is not a risk factor for worse symptom burden in patients with FM. Statin therapy should be offered to dyslipidemic FM patients with an appropriate medical indication to optimize their cardiovascular health.

Fibromyalgia (FM) is characterized by chronic widespread musculoskeletal pain, joint stiffness, and other systemic symptoms including mood disorder, cognitive dysfunction, sleep issues, and fatigue.^1^ It is a prevalent disorder, and estimates report more than 5 million Americans with a diagnosis of FM.^2^, ^3^, ^4^, ^5^ The etiology and pathophysiology of FM remain unclear, and there are limited data describing risk factors that may explain the correlation between FM and worsening symptom severity.^6^, ^7^, ^8^

The 3-hydroxy-3-methylglutaryl coenzyme A reductase inhibitors, also known as statins, are the most widely used medications to lower low-density lipoprotein cholesterol concentration and to decrease the risk for adverse cardiovascular events.^9^ However, concerns about the adverse effect profile of statin therapy exist, namely, statin-induced myopathy, which comprises muscle pain and tenderness, fatigue, nocturnal cramping, and tendon pain that tends to be generalized, affects proximal muscles, and worsens with exercise.^10^

Fibromyalgia is manifested with widespread musculoskeletal pain and tender points, and it is unclear whether statin therapy may further exacerbate preexisting musculoskeletal pain and tender points in patients with FM. A large-sample observational study reported that patients with FM-like symptoms are 3 times more likely to have statin-associated muscle complaints compared with those who do not have FM.^11^ Furthermore, it is well known that a structured exercise program is associated with improvements in physical, emotional, and social functioning in patients with FM.^12^ However, studies have reported that as many as 25% of patients who take statin therapy and exercise regularly may experience muscle complaints as an adverse effect.^13^ Thus, Mascitelli et al^14^ proposed that statin therapy may interfere with implementation of an exercise regimen in patients with FM that may lead to further exacerbation of their symptoms.

The objective of our prospective questionnaire study was to compare symptom severity between statin users and statin nonusers in a cohort of patients with FM, which to date no study has addressed. In addition, we sought to investigate the association of statin use with other secondary outcomes, including fatigue, cognitive dysfunction, mood disorders, and sleep problems. We hypothesized that preexisting diffuse musculoskeletal pain may place patients with FM at increased risk for exacerbating musculoskeletal pain and worsening symptom burden with use of statin therapy.

## Methods

### Population of Patients

This study was approved by Mayo Clinic Institutional Review Board. This was a prospective questionnaire study that included 668 patients with FM who were evaluated at the Fibromyalgia Clinic within a tertiary referral center (Mayo Clinic, Rochester, Minnesota) between May 2012 and November 2013. The diagnosis of FM was made on the basis of the American College of Rheumatology 1990 criteria^15^ or 2010 criteria.^16^ Data for statin use were abstracted from the electronic medical record and involved at least 1 month or longer use of statin. Patients who were receiving statin therapy for less than 1 month were excluded from analysis.

### Outcomes of Interest

The primary outcomes included FM symptom severity as measured by the Revised Fibromyalgia Impact Questionnaire (FIQ-R) and tender point count. The FIQ-R consists of 21 questionnaire items that assess FM symptom severity, functional status, and overall impact of FM. Each item is graded on a scale of 0 to 10, with weighted summary scores ranging from 0 to 100. Higher scores on the FIQ-R designate more severe symptom burden. Specifically, scores of 0 to less than 39, 39 to less than 59, and 59 to 100 designate mild, moderate, and severe symptom burden, respectively.^17^

Secondary outcome measures included fatigue, cognition, anxiety, depression, and sleep problems. The Multidimensional Fatigue Inventory 20-item questionnaire (MFI-20) evaluates fatigue in 5 subscales including general fatigue, physical fatigue, reduced activities, reduced motivation, and mental fatigue.^18^ Each subscale ranges between 4 and 20, with higher MFI-20 scores representing greater fatigue. The Multiple Ability Self-Report Questionnaire (MASQ) consists of 38 items that measure 5 domains related to cognition: language, visuoperceptual, verbal memory, visual memory, and attention.^19^ For each domain, scores range from either 0 to 30 or 0 to 40, with a total score range between 0 and 190. Higher scores represent more perceived cognitive dysfunction. The Generalized Anxiety Disorder 7-item (GAD-7) scale assesses anxiety; each item is scored on a scale of 0 to 3, with a total score range between 0 and 21.^20^ Scores of 5, 10, and 15 indicate mild, moderate, and severe anxiety, respectively. The Patient Health Questionnaire 9-item (PHQ-9) assesses depression; each item is scored on a scale of 0 to 3, with a total score range between 0 and 27.^21^ A total score of 10 or higher represents major depressive symptoms. The Medical Outcomes Study Sleep Problems Index II (SPI-II) consists of 9 items that measure sleep quality, with higher scores indicating poorer sleep quality.^22^

### Statistical Analyses

Outcomes were summarized by reporting mean and standard deviations for continuous outcomes and frequency (percentage) for categorical outcomes. We constructed both unadjusted and adjusted linear regression models to compare outcomes of interest between statin users and statin nonusers. Adjusted regression analysis was adjusted for age, sex, body mass index (BMI), ethnicity, current tobacco use, current opioid use, and current neuropathic medication use. A *P* value of less than .05 was considered to be significant. All analyses were performed using SPSS Statistics for Windows, version 21.0 (IBM Corp).

## Results

### Demographic Characteristics

A total of 668 patients with a mean age of 47.0±13.0 years were surveyed. There were 79 FM patients (11.8%) who were statin users, whereas 589 FM patients reported no current statin use (88.2%). Demographic and baseline characteristics are displayed in Table 1. Baseline characteristics were similar between statin users and the control group, except for 2 covariates. There was an association of older age (*P*<.001) and higher BMI (*P*=.001) in statin users compared with control patients.

**Table 1:** Demographic and Clinical Characteristics Based on Statin Use^a^

Characteristic	Overall cohort	Statin use		*P* value
	N=668	No (n=589)	Yes (n=79)	
Patient age (y)	47.0±13.0	46.2±12.9	55.0±11.3	<.001^b^
Sex				
Female	606 (90.7)	537 (91.2)	69 (87.3)	.299
Male	62 (9.3)	52 (8.8)	10 (12.6)	
Body mass index (kg/m^2^)	30.2±7.7	29.8±7.7	33.0±7.0	.001^b^
Ethnicity				
White	596 (89.2)	521 (88.4)	75 (94.9)	.720
Non-White	72 (10.8)	68 (11.5)	4 (5.1)	
Current tobacco use	94 (14.1)	82 (13.9)	12 (15.2)	.732
Current opioid use	202 (30.2)	180 (30.6)	22 (27.8)	.696
Current neuropathic medication use	322 (48.2)	281 (47.7)	41 (51.9)	.549

a Categorical variables are presented as number (percentage). Continuous variables are presented as mean ± standard deviation.

b *P*≤.05.

### Outcomes

Table 2 demonstrates primary outcomes of interest. Both unadjusted analysis and adjusted linear regression models revealed no association between statin use status and total FIQ-R scores (57.7±18.3 vs 59.0±18.1; adjusted β coefficient, −0.4; 95% CI, −4.8 to 4.1; *P*=.871). There were also no significant differences in separate FIQ domains of function, overall impact, and symptoms. Similarly, both unadjusted analysis and adjusted linear regression models revealed no association between statin use status and tender point count (14.8±4.1 vs 14.5±4.2; adjusted β coefficient, 0.2; 95% CI, −0.8 to 1.2; *P*=.732). Secondary outcomes of interest are displayed in Table 3. Both unadjusted and adjusted linear regression analysis did not reveal any difference in fatigue, cognition, anxiety, depression, or sleep problems between statin users and statin nonusers.

**Table 2:** Association Between Statin Use and FIQ Scores and Tender Points^a^

	Statin use		Unadjusted		Adjusted^b^	
	No	Yes	β coefficient (95% CI)	*P* value	β coefficient (95% CI)	*P* value
Total FIQ score	59.0±18.1	57.7±18.3	−1.3 (−5.7 to 3.1)	.568	−0.4 (−4.8 to 4.1)	.871
FIQ domains						
Function	14.9±7.4	14.6±7.1	−0.3 (−2.1 to 1.4)	.720	−0.4 (−2.2 to 1.4)	.652
Overall impact	12.6±5.5	12.1±5.6	−0.6 (−1.9 to 0.7)	.356	0.0 (−1.3 to 1.4)	.967
Symptoms	31.4±7.9	31.0±8.0	−0.4 (−2.3 to 1.5)	.664	0.0 (−1.9 to 1.9)	.990
Tender point count	14.5±4.2	14.8±4.1	0.3 (−0.6 to 1.3)	.503	0.2 (−0.8 to 1.2)	.732

a FIQ, Fibromyalgia Impact Questionnaire.

b Adjusted for age, sex, body mass index, ethnicity, current tobacco use, current opioid use, and current neuropathic medication use.

**Table 3:** Statin Use and Secondary Outcome Measures^a^

	Statin use		Unadjusted		Adjusted^b^	
	No	Yes	β coefficient (95% CI)	*P* value	β coefficient (95% CI)	*P* value
MFI-20 (fatigue)	75.9±13.8	74.3±14.9	−2.4 (−5.7 to 1.0)	.169	−1.8 (−5.2 to 1.7)	.319
MASQ (cognition)	95.9±22.9	96.4±23.2	−0.4 (−5.8 to 5.0)	.878	2.0 (−3.5 to 7.4)	.482
GAD-7 (anxiety)	8.4±6.0	9.2±5.6	0.8 (−0.6 to 2.3)	.266	1.4 (−0.1 to 2.9)	.070
PHQ-9 (depression)	12.2±5.7	12.1±6.3	−0.2 (−1.5 to 1.2)	.819	0.2 (−1.2 to 1.6)	.818
SPI-II (sleep problems)	58.3±19.3	54.4±18.6	−3.8 (−8.3 to 0.66)	.095	−0.1 (−4.5 to 4.4)	.981

a GAD-7, Generalized Anxiety Disorder 7-item; MASQ, Multiple Ability Self-Report Questionnaire; MFI-20, Multidimensional Fatigue Inventory 20-item; PHQ-9, Patient Health Questionnaire 9-item; SPI-II, Sleep Problems Index II.

b Adjusted for age, sex, body mass index, ethnicity, current tobacco use, current opioid use, and current neuropathic medication use.

## Discussion

We observed no difference in symptom severity and tender point count in patients with FM who were statin users vs patients with FM who did not take statin therapy. Concordant with this observation, there were also no differences in other secondary outcomes, including fatigue, cognition, mood, and sleep issues. This highlights that patients with FM, a population that already experiences generalized musculoskeletal pain and diffuse tender points, may not be at increased risk of experiencing worse muscle tenderness, increased tender points, or other detrimental clinical outcomes after statin therapy administration. Studies have estimated that more than 40% of patients who have a medical indication for statin therapy do not receive this therapy, and many patients refuse therapy because of concerns of muscle-related pain and adverse effects.^23^^,^^24^ The authors query whether this fear of adverse effects may be heightened in patients with FM who are already prone to diffuse musculoskeletal pain. However, on the basis of the results of this study, this concern may not be warranted and importantly may represent a significant barrier to optimization of cardiovascular health in dyslipidemic patients with FM.

The cause of statin-induced myopathy remains unclear. One proposed mechanism is that impaired cholesterol synthesis may lead to detrimental changes in the myocyte membrane.^25^ Another mechanism is that impaired cholesterol synthesis may lead to decreased coenzyme Q10 levels, which in turn may cause impaired mitochondrial enzyme activity and resultant myopathy.^26^ Finally, another potential mechanism is that statins may deplete isoprenoids, which are implicated in preventing myofiber apoptosis.^27^ Whereas evidence is limited on risk factors of statin-induced myopathy, some studies have proposed advanced age, female sex, impaired hepatic and renal metabolism, low BMI, alcohol use, surgery, trauma, and dietary effects.^10^^,^^28^^,^^29^

The strengths of this study include a prospective questionnaire design that used regression analysis to adjust for potential confounders. The only significant differences in baseline characteristics were older age and higher BMI in statin users. This is an expected finding as hypercholesterolemia is more likely as one gets older and gains more weight.^30^^,^^31^ Thus, we controlled for age and BMI as well as for other potential confounders when we performed adjusted regression analysis. Furthermore, we used a comprehensive set of validated questionnaires commonly used to assess clinical outcomes in patients with FM.

Larger scale prospective studies are warranted. Future studies should assess whether duration of statin therapy plays a role in muscle-related complaints. Furthermore, a dose-response relationship may also help inform appropriate dosing. Similar studies in the future should also clarify whether patients in the nonstatin cohort had an indication for statin therapy but did not take statin therapy. Our study did not stratify patients on the basis of the type of statin medication they received. The incidence of statin myopathy may vary by the type of administered statin. For example, only 5.1% of patients taking fluvastatin experience statin myopathy, whereas higher rates of statin myopathy are reported with administration of simvastatin (18.2%), atorvastatin (14.9%), and pravastatin (10.9%).^24^ Finally, it is well known that an exercise program may improve physical, emotional, and social functioning in patients with FM.^12^ However, exercise may also increase the risk for statin myopathy in those taking statin medication.^13^ Thus, future research efforts should clarify the role of exercise in patients with FM who take statin medications.

Several limitations are notable. The sample size was small for statin users (n=79), introducing the possibility of false-negative associations. Statin use status was analyzed as a binary variable, and no dose-response relation was analyzed. Importantly, we selected the statin cohort to include patients who took statins for at least 1 month. Typically, patients are more likely to develop short-term adverse effects of muscle pain and myalgias in the first few weeks, which may lead to discontinuation of statin therapy. This introduces selection bias because the study does not capture patients who stopped statin therapy after taking it for less than 1 month. Pertaining to the nonstatin cohort, our data collection strategy had limitations because we did not abstract details of patients who were previously receiving statin therapy but did not take statin therapy at the time of data abstraction. Finally, outcome measures were assessed by self-report questionnaires, which may be affected by reporting bias.

## Conclusion

Patients with FM who are receiving statin therapy for at least 1 month experience similar symptom severity, tender point count, fatigue, cognition, mood, and sleep issues compared with statin nonusers. This highlights that patients with FM, a population that already experiences generalized musculoskeletal pain and diffuse tender points, may not be at increased risk of experiencing worse muscle-related adverse effects. Statin therapy should be offered to dyslipidemic patients with FM with an appropriate medical indication to optimize their cardiovascular health.
